# Sequence analysis for the complete proviral genome of subgroup J Avian Leukosis virus associated with hemangioma: a special 11 bp deletion was observed in U3 region of 3'UTR

**DOI:** 10.1186/1743-422X-8-158

**Published:** 2011-04-08

**Authors:** Min Shi, Mingxing Tian, Cheng Liu, Yang Zhao, Yan Lin, Nianli Zou, Ping Liu, Yong Huang

**Affiliations:** 1 College of Veterinary Medicine, Sichuan Agricultural University, Ya'an, Sichuan, 625014, China; 2 Key Laboratory of Animal Disease and Human Health of Sichuan Province, Sichuan Agricultural University, Ya'an, 625014, China

## Abstract

**Background:**

Avian Leukosis virus (ALV) of subgroup J (ALV-J) belong to retroviruses, which could induce tumors in domestic and wild birds. Myelocytomatosis was the most common neoplasma observed in infected flocks; however, few cases of hemangioma caused by ALV-J were reported in recent year.

**Results:**

An ALV-J strain SCDY1 associated with hemangioma was isolated and its proviral genomic sequences were determined. The full proviral sequence of SCDY1 was 7489 nt long. Homology analysis of the env, pol and gag gene between SCDY1 and other strains in GenBank were 90.3-94.2%, 96.6-97.6%, and 94.3-96.5% at nucleotide level, respectively; while 85.1-90.7%, 97.4-98.7%, and 96.2-98.4% at amino acid level, respectively. Alignment analysis of the genomic sequence of ALV-J strains by using HPRS-103 as reference showed that a special 11 bp deletion was observed in U3 region of 3'UTR of SCDY1 and another ALV-J strain NHH isolated from case of hemangioma, and the non-functional TM and E element were absent in the genome of SCDY1, but the transcriptional regulatory elements including C/EBP, E2BP, NFAP-1, CArG box and Y box were highly conserved. Phylogenetic analysis revealed that all analyzed ALV-J strains could be separated into four groups, and SCDY1 as well as another strain NHH were included in the same cluster.

**Conclusion:**

The variation in envelope glycoprotein was higher than other genes. The genome sequence of SCDY1 has a close relationship with that of another ALV-J strain NHH isolated from case of hemangioma. A 11 bp deletion observed in U3 region of 3'UTR of genome of ALV-J isolated from case of hemangioma is interesting, which may be associated with the occurrence of hemangioma.

## Background

Avian Leucosis virus subgroup J (ALV-J) was first reported in 1989 from commercial meat-type chickens with myelocytomatosis [1]. It is a novel subgroup different from classical ALV subgroup A, B, C, D and E, and a recombinant between exogenous avian leukosis viruses (ALVs) and the endogenous avian retrovirus (EAV) family of EAVs [2,3]. ALV-J could be transmitted from broiler breeders to progeny more frequently than other ALV subgroups by Vertical transmission [4], and has caused severe economic losses in breeding farms all over the world. Recent studies showed that the host range of ALV-J had been largely widened, it could infect not only broilers but also commercial layers. Meanwhile, some new clinical pathotypes were observed in diseased flocks, such as hemangioma, which might be observed on the skin of the trunk, digitus, joint, wing and internal organs.

In China, the type of neoplasma induced by ALV-J was mainly myeloma leucosis (ML), and it occurred mainly in white meat-type chickens [5]. However, few cases of hemangioma caused by ALV subgroup J were reported in recent years [6,7], and they were diagnosed mainly by histopathological examination and PCR detection. To updates, two complete proviral genomic sequences of ALV-J strain associated with hemangioma were reported, which were NHH (HM235668) and JL0901 (not published in NCBI).

In 2009, an infectious disease occurred in a grandparent breeding farm in Sichuan province of China, characteristics mainly of weakness, inappetance and emaciation. Hemangioma was observed in the digiti of the sick. The gross pathology seen in organs of diseased chickens included hepatomegaly, splenomegaly and gray-white nodules of various sizes on the surface of the liver, but no myeloma leucosis was observed. In this study, an ALV-J strain SCDY1 was isolated from the blood samples of the diseased chickens, and its complete genome sequence was amplified and analyzed, which should be helpful for the research on the pathogenesis of hemangioma induced by ALV-J.

## Methods

### Chicken embryo fibroblast (CEF)

Specific-pathogen-free (SPF) eggs were obtained from the Beijing experimental animal center (Merial Inc., Beijing, China) and hatched at our lab. Ten-day-old chicken embryos were used to prepare the Chicken embryo fibroblast (CEF) cell cultures.

### Virus isolation

The whole blood sample was inoculated onto the CEF monolayers prepared from 11-day-old embryonated eggs, and incubated at 37°C with 5% CO_2 _for five days for each passage. Uninfected CEF monolayers were used as negative control. After one blind passage, the existence of ALV-J in CEF was verified by PCR detection of 545 bp repeated sequence. Three blind passage were conducted until the result of PCR detection of cellular genome and RT-PCR detection of supernatant were both positive.

### Primers

First, a pair of primers designed by smith [8] was synthesized and used to detect the proviral genomic DNA of ALV-J in infected CEF, or the viral RNA of ALV-J in the supernatant of infected CEF. Next, nine pairs of primers (Table 1) were designed according to the published sequences of HPRS-103 to amplify nine fragments which overlapped with each other and covered the whole genome of ALV-J. The ninth pair was used specifically to amplify the LTRs of circular DNA of ALV-J.

**Table 1:** Primers used to amplify the proviral genomic DNA of SCDY1

**No**.	Name	Sequence(5'----3')	Position corresponding to the genome of HPRS-103	Expected size(bp)
1	A1F	GGCTCTTATGTAACGATG	16-33	614
	A1R	GGAAATCACCTTTATGACGG	610-629	
2	A2F	GATGTGATAGTTAGGGAATAGTGG	336-359	1160
	A2R	CTCTCATTAGATTCGTAACGTC	1474-1495	
3	A3F	TCACAAGACTGGCTGATACGGT	1376-1397	1098
	A3R	CCGAAATAATAGTGATGTCCGC	2452-2473	
4	A4F	TCATTCTGACTAACACTGGGAG	2378-2399	1191
	A4R	CGCAACTGCTCATAAAAGGG	3548-3568	
5	A5F	GTAGCAGAACCCAGGATAGC	3447-3466	1376
	A5R	GTGAAGCAGGACCCATTATC	4803-4822	
6	A6F	CGTGTCACATCGGTTGCTG	4725-4743	745
	A6R	CCCTGGGACAACGGAAATA	5451-5469	
7	A7F	ACTCTAAGAAGAAGCCGCC	5342-5360	1921
	A7R	CGA TTCGCAAGT TTCATACC	7243-7262	
8	A8F	GTTTGCCTTGCTTGTCATAG	6855-6874	968
	A8R	CAGGTGCTCGTAGTTGTCA	7804-782	
9*	A9F	TTAGGAAGGCAACAGACGG	7644-7662	466
	A9R	GGGCGACCAGAATCACG	574-591	

* Primers used to amplify the long terminal repeat (LTR) sequences from circularized viral DNA of SCDY1.

### Genomic DNA extract and PCR amplification

The total DNA was extracted from chicken embryo fibroblast (CEF) infected with virus by using a sodium dodecyl sulfate (SDS) - proteinase K and phenol/chloroform/isoamylol (25:24:1) protocol. Genomic DNA PCR amplification was performed according to the manual of rTaq kit (Takara, Dalian, China), by using the proviral genomic DNA as template. Primers pairs 1-8 were used to amplify the internal region of SCDY1 genome; while primer pair 9 was used to amplify the LTRs of circular DNA by using total DNA of CEF infected by SCDY1 for 8-24 h as templates. The optimum conditions for PCR were as follows: 94°C for 4 min, ten cycles at 94°C for 35 s, 55°C for 35 s (a decrease of 0.5°C per cycle), 72°C for 40 s and followed by 20 cycles at 94°C for 35 s, 50°C for 35 s, 72°C for 40 s and a final elongation at 72°C for 10 min. The PCR product was analyzed in 1% agarose in Tris-borate-EDTA (TBE) buffer gel containing 0.5 mg/ml ethidium bromide.

### Cloning and sequencing of proviral genomic DNA of SCDY1

The PCR products were isolated from agarose gels and purified using E.Z.N. ATM Gel Extraction Kit (TaKaRa, Japan), then cloned into pMD19-T vector (Takara, Dalian) and transformed into DH5α E. Coli competent cell. Confirmation of clones containing recombinant plasmid was achieved by PCR and restriction enzyme (RE) digestion. The recombinant plasmid was sequenced by Shanghai Sanggong Biologiucal Engineering Tachnology & Services Co., Ltd (shanghai, China).

### Sequence analysis

The complete genome sequences of the isolate was compared with these of other ALV-J strains published in GenBank, including Chinese strains isolated from white broilers and commercial layers by using the Clustal W method in MegAlign program of the DNASTAR package. Transcriptional regulatory elements in U3 were analyzed by the online service system of NSITE (Recognition of Regulatory motifs) of Soft Berry (http://linux1.softberry.com/berry.phtml). Phylogenetic analysis of the complete proviral sequences of ALV-J was performed with the neighbor-joining method using MEGA version 4.0. The background of the reference strains used in this study and their accession numbers are listed in table 2.

**Table 2:** ALV-J reference strains used in this study

ALV-J strains	Origin			Tumor Phaenotype	Accesstion number
	year	country	host		
HPRS-103	1995	UK	White broiler	ML	Z46390
YZ9902	1999	China	White broiler	ML	HM235670
ADOL-7501	2001	USA	White broiler	N/A	AY027920
NX0101	2001	China	White broiler	ML	DQ115805
NM2002-1	2002	China	White broiler	ML	HM235669
JS-nt	2003	China	White broiler	ML	HM235667
SD07LK1	2007	China	Commercial layer	ML	FJ216405
NHH	2007	China	Commercial layer	Hemangioma	HM235668
HN*	2007	China	Commercial layer	Hemangioma	HM235666
HAY013	2008	China	Local "yellow"	ML	HM235665
SCDY1	2009	China	Grandparent breed	Hemangioma	HQ425636
JS09GY3	2009	China	Commercial layer	Hemangioma and ML	GU982308
JS09GY6	2009	China	Commercial layer	Hemangioma and ML	GU982310

* isolate with no complete genomic sequence. ML means myeloma leukosis; ^*N/A *^data not available

## Results

### Virus isolation and identification

An ALV-J strain was isolated from the sampled blood, and was designated as SCDY1. The genomic DNA of infected CEF were positive in PCR detection, and the supernatant of infected CEF was positive in RT-PCR detection (data not shown), indicating the existence of proviral genomic DNA of ALV-J in infected CEF and ALV in the supernatant of infected CEF.

### PCR amplification of proviral genome

Total DNA from SCDY1-infected CEF was used as PCR templates, and nine DNA fragments with the expected sizes of 0.614, 1.16, 1.098, 1.191, 1.376, 0.745, 1.921, 0.968, 0.466 kb long were amplified (data not shown).

### Sequence analysis of the proviral genome of SCDY1

**Figure 1:**
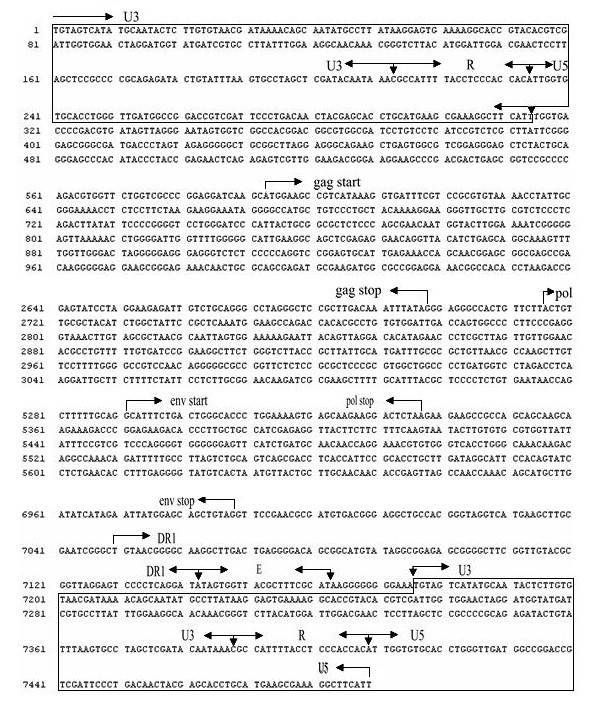
**The structure of complete proviral genome of SCDY1**. The boxed sequence at the two ends shows 5'LTR and 3'LTR. The arrows show the beginning and stopping nucleotides for each ORF or principal fragments DR1/E element. Partial sequences of SCDY1 genome were not shown.

Sequences of the PCR products were aligned by using the EDITSEQ program in DNAstar software (DNASTAR Inc., Madison, WI 53715, USA). The complete proviral genome sequence of SCDY1, compiled from sequences of nine overlapping DNA fragments, was 7489 nt in length, and submitted to GenBank under accession No. HQ425636. The structure of SCDY1 proviral genome was presented in Figure 1.

Comparisons of the long terminal repeat (LTR) sequence showed that the LTR of SCDY1 shared a homology of 89.8-92.7% with these of other reference strains, and the LTR sequences of strains HPRS-103 and HN had the highest homology with these of SCDY1 (Table 3).

**Table 3:** The Homology of nucleotide sequences of LTR, gag, pol and env genes between SCDY1 and other reference strains

ALV-J strains*	genes or fragment of SCDY1			
Name	LTR	gag	pol	env
HPRS-103	92.7	96.3	97.3	94.2
YZ9902	90.1	95.5	97.2	93.6
ADOL-7501	90.4	94.8	97.4	90.3
NX0101	90.4	94.3	97.4	92.9
NM2002-1	91.1	95.7	96.9	92.7
JS-nt	91.7	96.0	96.9	92.9
SD07LK1	89.8	96.2	97.6	93.7
NHH	91.4	96.5	96.6	94.2
HN^	92.7	---	---	---
HAY013	89.8	95.5	97.1	93.6
JS09GY3	91.1	95.3	97.3	93.8
JS09GY6	90.8	95.2	97.1	94.0

* pathotype of isolates was available in table2; ^ means strain with only LTR sequence was available; "---" means no result could be obtained.

Comparisons of the three major structural genes revealed that the homology of nucleotide sequence of env, pol and gag gene between SCDY1 and other isolates in GenBank were 90.3-94.2%, 96.6-97.6% and 94.3-96.5%, respectively, and the homology of amino acid sequence of env, pol and gag gene between SCDY1 and other isolates in GenBank were 85.1-90.7%, 97.4-98.7% and 96.2-98.4%, respectively. The gag and pol genes of ALV-J were more conservative than the env gene relatively (Table 3, 4).

**Table 4:** The Homology of amino acid sequences of gag, pol and env proteins between SCDY1 and other reference strains

ALV-J strains*	protein of SCDY1		
	gag	pol	env
HPRS-103	97.2	98.7	90.3
YZ9902	97.0	98.3	89.5
ADOL-7501	96.2	98.2	85.1
NX0101	94.3	97.7	89.0
NM2002-1	97.0	98.2	89.3
JS-nt	97.4	98.2	89.7
SD07LK1	97.3	98.2	89.6
NHH	98.4	97.4	90.7
HAY013	96.6	98.3	90.5
JS09GY3	96.7	98.4	89.1
JS09GY6	96.6	98.3	89.1

* pathotype of isolates was available in table2.

**Figure 2:**
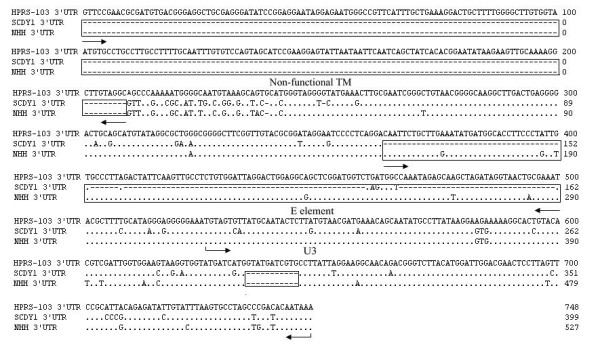
**Comparison of the 3'UTR nucleotide sequences for different ALV-J strains**. "---" means the deletion of nucleotide.

**Figure 3:**
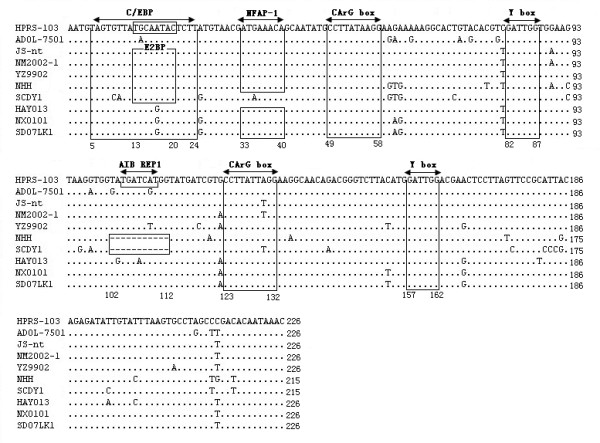
**Transcriptional regulatory elements in U3 of different ALV-J strains**. Bases deletions showed as "---" and nucleotide same to HPRS-103 indicated as "."; motifs were indicated in panes.

Homology analysis based on 3'UTR fragment of ALV-J associated with hemangioma was made by Clustal W method in MegAlign, and prototype strain HPRS-103 was used as a reference. The result showed that SCDY1 and NHH shared two almost identical deletions in 3'UTR, Which were the deletion of most part of the non-functional TM and a special 11 bp deletion in U3 region. Furthermore, most parts of E element in SCDY1 genome were absent; while, it was not observed in that of NHH (Figure 2). Online service analysis system of NSITE of SoftBerry showed that the transcriptional regulatory elements in U3 region of SCDY1 genome contained C/EBP, E2BP, CArG box and Y box, and they were highly conserved when comparing to these of the HPRS-103 strain. This special 11 bp deletion was found in the upstream of CArG box (Figure 3) and the sequence "TGATCAT" observed in this special 11 bp deletion region was recognized as a transcriptional regulatory element ABI REP1, which was the regulatory factor of human gene Apo-AI.

### Phylogenetic relationships between SCDY1 and other ALV-J strains

**Figure 4:**
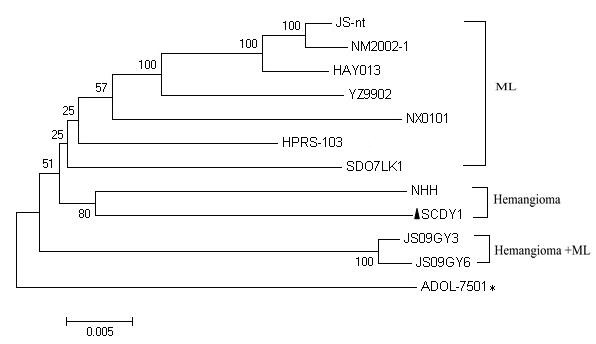
**Phylogenetic tree of complete genome sequences of ALV-J with different clinical phaenotype**. Black triangle (▲) referred to isolate SCDY-1, * referred to the isolate with unknown tumor phaenotype.

Phylogenetic analysis of the complete genome sequences of SCDY1 and other eleven ALV-J strains demonstated that SCDY1 and other eleven reference strains could be grouped into four clusters, SCDY1 and another strain NHH isolated from case of hemangioma were in the same cluster (Figure 4).

## Discussion

ALV-J has spread all over the world and caused severe economic losses in broiler breeder flocks since it was isolated [5,9-11]. It can infect all species of chicken, but the types of neoplasma and the morbidity vary in different cases. The most common neoplasma observed in infected flocks was myeloid leucosis (ML) [12]; however, myelocytomas are not exclusive to ALV-J, other types of neoplasma such as histiocytic sarcomatosis, feather abnormalities, myelocyte infiltration in bones, hemangioma has also been reported [13-16]. Hemangioma has been even the most common neoplasm in broiler breeders in the UK [17], and an outbreak of hemangioma with 20% mortalities in young white Leghorn layers in Israel has also been reported [18]. In China, hemangioma was observed in several commercial layer flocks infected by ALV-J during 2007-2009 [19].

In this paper, a field ALV-J strain named as SCDY1 was isolated and its the complete proviral genome nucleotide sequence was determined. It contains 7489 nt with no EcoR I site, which could be found in the genome of NHH strain isolated from case of hemangioma. Homology analysis of the genes of SCDY1 and other reference strains showed that the envelope gene of ALV-J was prone to evolve compared to gag and pol genes, as the identity of the env gene between strain SCDY1 and other strains ranged from 90.3% to 94.2% at the nucleotides level and from 85.1% to 90.7% at the amino acid level respectively, lower than these of gag and pol genes.

The 3'UTR of avian retroviruses is important in viral pathogenesis and replication, the LTR enclosed within the 3' UTR contains powerful transcription regulatory elements that might differ among viruses and determine the virulence [20]. According to sequences analysis, the transcriptional regulatory elements of U3 region of SCDY1 genoem contained several transcriptional elements such as C/EBP, E2BP, CArG box and Y box, which played an important role in the efficient transcription of virus; while, NFAP-1 was not observed in SCDY1 genome, and it could be found in these of other analyzed strains. NFAP-1 could be recognized by the activator protein 1 (AP-1), and AP-1 is a heterodimeric protein composed of proteins belonging to the c-Fos, c-Jun, ATF and JDP families. It regulates gene expression in response to a variety of stimuli, including cytokines, growth factors, stress, and bacterial and viral infections [21]. The absent of NFAP-1 might affect the infection abilities of ALV-J virus.

Most of non-functional TM and E element of SCDY1 in 3'UTR were absent, which shared identical deletions with some ML strains isolated from meat-type chickens; while, the deletion of E element in SCDY1 genome was not observed in the genome of another strain NHH isolated from case of hemangioma. The E element has been found exclusively in some of the sarcoma viruses and ALV-J [22-24]. It may play a role in oncogenesis based on its close association with the src gene; however, it seemed not to be essential for oncogenesis, because other related oncogenic avian retroviruses lack the E element [25]. Therefore, the pathogenicity of SCDY1 may be unaffected by the deletion of TM and E element. The occurrence of hemangioma may be associated with multiple factors such as genetic backgroud, environment, concomitant infections, immunocompetence and the mode of transmission.

Interestingly, a special 11 bp deletion was observed in the U3 region of ALV-J strains associated with hemangioma. Online service analysis showed that a sequence "TGATCAT" within this special 11 bp region of U3 of ALV-J strains other than SCDY1 was a transcriptional regulatory element ABI REP1, which was the regulatory factor of human gene Apo-AI and function in the process of recognition of regulatory motifs. One binding factor of ABI REP1 named as HNF-4 was also found, and it served as an inhibitor modulator and played an important role in the regulation of eukaryotic genes. Although the special 11 bp deletion did not locate in CArG box, PRE box or Y box, its appearance may played a role in the transcriptional regulation of ALV-J genes, further study such as reverse genetics should be taken to test its function in oncogenesis.

So far, there are few proviral genomic sequence of ALV-J strains associated with hemangioma have been reported, the sequence comparison and phylogenetic analysis of SCDY1 and other ALV-J strains may help to reveal the evolution rhythm of ALV-J strains prevalent in fields, and benefit for the research on the pathogenicity and biological characteristics of ALV-J strain.

## Competing interests

The authors declare that they have no competing interests.

## Authors' contributions

MS: carried out most of the experiments and wrote the manuscript, YH: carried out study design, and revised the manuscript. MXT, CL, YZ, YL, NLZ, PL, XTW, SJC helped in experiments. All authors read and approved the final manuscript.
